# The Effects of Acidosis on eNOS in the Systemic Vasculature: A Focus on Early Postnatal Ontogenesis

**DOI:** 10.3390/ijms23115987

**Published:** 2022-05-26

**Authors:** Dina K. Gaynullina, Olga S. Tarasova, Anastasia A. Shvetsova, Anna A. Borzykh, Rudolf Schubert

**Affiliations:** 1 Department of Human and Animal Physiology, Faculty of Biology, M.V. Lomonosov Moscow State University, 119234 Moscow, Russia; dina.gaynullina@gmail.com (D.K.G.); ost.msu@gmail.com (O.S.T.); anastasiashvetsova92@gmail.com (A.A.S.); borzykh.anna@gmail.com (A.A.B.); 2 Laboratory of Exercise Physiology, State Research Center of the Russian Federation-Institute for Biomedical Problems, Russian Academy of Sciences, 123007 Moscow, Russia; 3 Physiology, Institute of Theoretical Medicine, Faculty of Medicine, University of Augsburg, 86159 Augsburg, Germany

**Keywords:** nitric oxide, eNOS, anticontractile influence, endothelium, acidosis, pH, early postnatal development, vasculature

## Abstract

The activity of many vasomotor signaling pathways strongly depends on extracellular/intracellular pH. Nitric oxide (NO) is one of the most important vasodilators produced by the endothelium. In this review, we present evidence that in most vascular beds of mature mammalian organisms metabolic or respiratory acidosis increases functional endothelial NO-synthase (eNOS) activity, despite the observation that direct effects of low pH on eNOS enzymatic activity are inhibitory. This can be explained by the fact that acidosis increases the activity of signaling pathways that positively regulate eNOS activity. The role of NO in the regulation of vascular tone is greater in early postnatal ontogenesis compared to adulthood. Importantly, in early postnatal ontogenesis acidosis also augments functional eNOS activity and its contribution to the regulation of arterial contractility. Therefore, the effect of acidosis on total peripheral resistance in neonates may be stronger than in adults and can be one of the reasons for an undesirable decrease in blood pressure during neonatal asphyxia. The latter, however, should be proven in future studies.

## 1. Acidosis Generally Reduces Tone of Arterial Vessels

The activity of many vasomotor signaling pathways strongly depends on pH. Therefore, intracellular pH is powerfully regulated by several membrane transporters, that remove excess acids or bases from the cell. In vascular cells, proton extrusion is accomplished mainly by Na^+^/HCO_3_^−^ cotransport and Na^+^/H^+^-exchange mechanisms, and excess HCO_3_^−^ is removed in exchange for Cl^−^ [1,2].

A lowered pH—acidosis—can develop due to the accumulation of proton-donating metabolites in the extracellular fluid of intensively working organs such as contracting skeletal muscles or because of renal, gastrointestinal, and metabolic disorders (so-called metabolic acidosis). In addition, the accumulation of CO_2_ in the blood due to respiratory disorders causes hypercapnic (respiratory) acidosis. In the course of several minutes, changes in extracellular pH are transmitted into changes in intracellular pH, which have the same direction and are equal to about a half or more of the extracellular change [1,3]. Acidosis can considerably affect the functioning of many organs, both directly and through the alteration of their blood supply. In addition, in some cases acidosis is associated with oxidative stress [4], which can be a supplementary factor affecting vascular functioning [5,6,7,8].

In some studies, extracellular acidosis was shown to increase arterial tone, as in the aorta and coronary arteries [9,10,11,12]. However, the prevailing effect of acidosis is the opposite. Generally, acidosis decreases vascular reactivity to constrictor stimuli (exerts an anticonstrictor effect) and potentiates vasodilatory responses. Importantly, such effects of acidosis are clearly manifested in small arteries and arterioles, which regulate peripheral vascular resistance, and hence the level of arterial blood pressure. Acidosis has been shown to decrease contractile responses of resistance arteries of various organs, including intestinal arteries [13,14], cerebral arteries [15,16,17], retinal arteries [18], mammary arteries [19], and skeletal muscle arteries [20]. The effects of acidosis on vascular tone can be realized through various mechanisms affecting both smooth muscle and endothelial cells.

Relaxation of arterial smooth muscle cells is associated with a decrease in the intracellular Ca^2+^ concentration [15,18]. This is often due to the depression in voltage-operated L-type Ca^2+^-channel activity [2,21], the opening probability of which directly depends on extracellular pH [22]. Further, low intracellular pH causes vasorelaxation via activation of ATP-sensitive K^+^ (K_ATP_) channels [23,24]. In addition, large-conductance Ca^2+^-activated K^+^-channels (BK_Ca_-channels) demonstrate a dual response to acidification: they are directly inhibited by low intracellular pH [25] but can be activated by acidosis-potentiated Ca^2+^-signals from the sarcoplasmic reticulum (Ca^2+^-sparks), at least in cerebral arterioles [26]. An increase in potassium conductance hyperpolarizes the vascular smooth cell membrane, thereby providing an additional mechanism for the decrease in voltage-operated Ca^2+^-channel activity and Ca^2+^-influx from the extracellular space. Finally, long-term depression of vasocontractile responses by acidosis is associated with a decrease in the Ca^2+^-sensitivity of the smooth muscle cell contractile apparatus due to the suppression of Rho-kinase activity [27]. Of note, the maximum of Rho-kinase activity is achieved at pH 7.4 and activity markedly decreases at pH < 7.0 [27].

Endothelium-dependent relaxation of vascular smooth muscle can be mediated by nitric oxide (NO), prostacyclin, and endothelium-derived hyperpolarizing factor (EDHF), which is a combination of several molecular mechanisms [28]. It was demonstrated that acidosis can increase the activity of the NO-signaling pathway in the vascular wall [13,23,24,29]. A potentiating effect of acidosis was suggested for the EDHF-pathway as well [29]. Contrarily, the contribution of prostacyclin to endothelium-dependent vasorelaxation may be decreased by acidosis [29].

In this review, we will focus on the functional effects of acidosis on the NO-signaling pathway in the vasculature, based on the key role of NO in vascular physiology and pathophysiology. Importantly, the activity of the NO-signaling pathway in the systemic vasculature is a key mechanism for the protectively low blood pressure in the circulatory system at perinatal age [30]. Therefore, in the last section of this review, we discuss the effects of acidosis on eNOS functional activity during early postnatal ontogenesis.

## 2. NO as a Key Vasorelaxing Factor in the Vasculature

Endothelial NO-synthase (eNOS) is the enzyme that produces NO—a key vasorelaxatory substance derived from the endothelium [31]. eNOS activity in vascular endothelial cells is regulated in many ways. The enzymatic activity of eNOS depends on its substrate L-arginine, the availability of cofactors (tetrahydrobiopterin (BH_4_) is an essential cofactor), and can be suppressed by endogenous inhibitors, in particular asymmetrical dimethyl arginine (ADMA) [31]. A key regulatory mechanism that affects eNOS activity is its dependence on intracellular Ca^2+^, determined by the activity of endothelial ion channels and calcium transporters located in particular in the cell membrane and the membrane of the sarcoplasmic reticulum [32]. Furthermore, the functional state of eNOS is controlled by the degree of its modifications at the transcriptional, post-transcriptional, and post-translational levels (for example, phosphorylation, palmitoylation, S-glutathionylation, and S-nitrosylation) [33,34]. Similar modifications of proteins involved in the regulation of eNOS activity will also affect eNOS function, albeit indirectly. This, however, is beyond the scope of this review. In addition, eNOS can interact with various proteins that are able to change eNOS activity, (e.g., caveolin-1, heat shock protein 90 (HSP90), endoglin, nitric oxide synthase trafficking (NOSTRIN), nitric oxide synthase-interacting protein (NOSIP), etc.) [34,35]. A detailed discussion of the regulation of eNOS activity including enzyme modifications, as well as the interaction of eNOS with other proteins, has been the subject of many previous review articles (see, for example, [33,34,35,36,37]). Therefore, it is not the aim of the authors of the present review to provide a comprehensive overview of the available literature on the regulation of eNOS in general. We briefly discuss only the mechanisms regulating eNOS activity that can potentially be affected by extracellular acidosis.

The Ca^2+^/calmodulin complex is an indispensable regulatory factor of eNOS. Ca^2+^ entry into endothelial cells is provided by different types of transient receptor potential (TRP) channels as well as by the Piezo1 channel [32,38]. Of note, the TRPV1 channel was suggested to function not only as a Ca^2+^ channel but also as a scaffold for the recruitment and formation of a complex comprising eNOS and key regulatory kinases, thereby providing necessary protein–protein interactions for the appropriate activation of eNOS [39]. Further, a functional complex between the TRPV4 channel and eNOS was described as important for proper endothelial functioning and vasodilation [40]. In addition, the amplification of Ca^2+^ signaling in endothelial cells may occur via Piezo1/Pannexin1-mediated ATP release, where the latter activates purinergic receptors thereby augmenting Ca^2+^ entry [32,38,41].

In addition to its dependency on Ca^2+^, there is another well-known regulatory mechanism of eNOS, phosphorylation. Thus, eNOS can be phosphorylated at several serine (Ser), threonine (Thr), and tyrosine (Tyr) residues, representing activatory and inhibitory phosphorylation sites, whereby phosphorylation/dephosphorylation may dynamically modulate eNOS enzyme activity. These phosphorylation sites include Tyr81, Ser114, Thr495, Ser615, Ser633, Tyr657, and Ser1177 [33,42].

*Ser1177*. This phosphorylation site is considered as one of the main eNOS activation sites [33,42,43]. In cultured endothelial cells, the level of phosphorylation of Ser1177 rapidly increases under the influence of shear stress [44] and receptor agonists, such as bradykinin or vascular endothelial growth factor [45]. The main pathway responsible for such phosphorylation is the PI3/Akt -kinase pathway [44]. Phosphorylation of this site is also possible following the action of Ca^2+^/calmodulin-dependent kinase II (CaMKII) [45], protein kinase A (PKA) [46], protein kinase G (PKG) [42,47] or protein kinase N2 [48]. In addition, it was found that the activation of AMP-activated protein kinase in endothelial cells also leads to an increase in the level of eNOS phosphorylation at this site and, accordingly, the production of nitric oxide [49,50,51,52].

*Ser615* and *Ser633*. The phosphorylation of these residues was shown to elevate eNOS activity and NO release [33,42,53,54]. The phosphorylation of Ser615 and Ser633 can be induced by Akt, PKA, AMPK, or Pim1 (proviral integration site for Moloney murine leukemia virus, serine/threonine kinase) [33,42,53,55,56,57]. In addition, Ser633 was shown to be phosphorylated by extracellular signal-regulated kinases ERK1/2 [58,59].

*Thr495*. Phosphorylation of this residue leads to a decrease in eNOS activity. The kinase that phosphorylates eNOS at Thr495 is claimed to be protein kinase C (PKC); PKC inhibitors considerably increase the synthesis of NO [45]. In addition, in cultured endothelial cells, this regulatory site was shown to be phosphorylated by Rho-kinase, since a Rho-kinase inhibitor reduced thrombin-induced eNOS phosphorylation at Thr495 [60].

*Ser114*. Controversial effects on eNOS enzymatic activity (activation in some studies, inhibition in others) were reported when eNOS was phosphorylated at this site (reviewed in [42]). Accordingly, the exact mechanisms and kinases responsible for this phosphorylation are not yet fully understood [42].

The role of tyrosine phosphorylation of eNOS seems to be less studied. Src kinase and Abelson tyrosine protein kinase (ABL)1 were shown to phosphorylate eNOS at Tyr81 and increase its enzymatic activity [33,42,61,62]. In contrast, phosphorylation at Tyr657 was shown to be mediated by proline-rich tyrosine kinase 2 (PYK2) and to decrease eNOS enzymatic activity [33,42,63,64,65].

Of note, the regulation of eNOS activity may also occur by phosphatases, in particular by vascular endothelial protein tyrosine phosphatase (VE-PTP), which demonstrates highly selective expression in endothelial cells [66]. VE-PTP can dephosphorylate eNOS at Tyr81 thereby decreasing its enzymatic activity [62,67]. Accordingly, VE-PTP inhibition increased the phosphorylation of eNOS at Tyr81 and NO production [62,67].

Thus, eNOS activity in endothelial cells can be dynamically regulated by phosphorylation/dephosphorylation under the action of various kinases, which provides fine-tuning of nitric oxide synthesis and, accordingly, its physiological effects on the cardiovascular system.

NO derived from endothelial cells diffuses into smooth muscle cells, which leads to vasorelaxation. The key acceptor of NO in smooth muscle is soluble guanylate cyclase, which produces cyclic guanosine monophosphate (cGMP) from guanosine-5’-triphosphate (GTP). cGMP activates PKG, which phosphorylates serine and threonine residues of a variety of targets in smooth muscle cells. The list of PKG targets in smooth muscle cells includes proteins involved in the regulation of the cytoplasmic Ca^2+^ concentration and the Ca^2+^-dependence of smooth muscle contraction.

One of the main targets of PKG is the BK_Ca_ channel, which is activated by PKG-dependent phosphorylation [68,69,70]. This results in hyperpolarization of smooth muscle cells, subsequent deactivation of voltage-gated calcium channels, and, accordingly, a decrease in Ca^2+^ entry into the cell. In addition, PKG can induce hyperpolarization by phosphorylation of other types of potassium channels in smooth muscle cells [71]. Further, PKG is able to phosphorylate phospholamban, leading to an increase in the activity of Ca^2+^-ATPase (SERCA 2A) and, consequently, to a decrease in the cytoplasmic Ca^2+^ concentration [72]. Moreover, PKG can phosphorylate IRAG proteins, a PKG substrate associated with receptors for inositol trisphosphate (Inositol trisphosphate receptor-associated cGMP-kinase substrate). IRAG is constitutively associated with PKG and inositol trisphosphate receptors in the sarcoplasmic membrane. Upon activation, PKG phosphorylates IRAG, which leads to suppression of Ca^2+^ release from the reticulum [72].

In addition to influencing the Ca^2+^ homeostasis of smooth muscle cells, PKG regulates the Ca^2+^ sensitivity of their contractile apparatus. PKG is able to phosphorylate and inactivate the RhoA protein thereby leading to a decrease in procontractile Rho-kinase activity. Second, PKG can phosphorylate the regulatory subunit of myosin light chain phosphatase at the Ser695 site, thereby increasing the activity of the phosphatase. In parallel with an increase in the level of phosphorylation at the Ser695 residue, there is a decrease in the level of phosphorylation at the inactivation site of Thr696 [73] which removes a block from the activity of myosin light chain phosphatase.

Thus, the overall relaxatory effect of NO in vascular tissue would depend not only on the level of NO production by the endothelium but also on the smooth muscle cell sensitivity to NO, which is determined by the expression level/activity of different proteins in the NO-signaling cascade in smooth muscle cells.

## 3. Acidosis Increases eNOS Functional Activity and Expression in Arteries

Effects of acidosis on eNOS were studied at different levels, including short-term effects on eNOS functional activity and long-term effects on eNOS gene expression.

### 3.1. Metabolic Acidosis

Metabolic acidosis induced by an extracellular pH decrease to 7.0 or 6.5 strongly potentiated NO synthesis in isolated aortic endothelial cells loaded with the DAF-FM fluorescent dye [24]. Further, extracellular acidification to 6.5 was shown to increase intracellular NO concentration in endothelial and smooth muscle cells in rat aorta [74].

Accordingly, metabolic acidosis increased eNOS functional activity in several studies on isolated arteries. Extracellular acidification to pH 7.0 enhanced the endothelium-dependent relaxation of the isolated aorta to acetylcholine [75]. In another study on rat aorta, extracellular acidosis-induced relaxation was considerably reduced by a NO-synthase inhibitor [24]. The vasorelaxing effect of metabolic acidosis can be associated at least in part with an augmented NO-sensitivity of smooth muscle cells, judged by an augmented cGMP content in aortic smooth muscle cells and larger responses of the endothelium-denuded aorta to NO-donors [75].

In the isolated rat mesenteric artery acidification of the external solution to pH 6.6 led to the reduction of contractile responses to electrical field stimulation of intramural sympathetic nerves as well as to exogenous noradrenaline [13]. These effects of acidosis were diminished in the presence of NO-synthase inhibitors in the case of nerve stimulation and abolished in the case of noradrenaline-induced contraction [13]. In another study, extracellular acidosis (pH 6.8) enhanced acetylcholine-induced relaxations of mesenteric arteries [29]. This increase in endothelium-dependent relaxation was mediated by the NO-signaling pathway [29]. Further, acidosis increased the level of NO metabolites (NOx) as well as the cGMP level in the aortic wall [29].

In rat middle cerebral arteries a stepwise decrease in the pH of the external solution from 7.4 to 6.0 also induced relaxatory responses [23]. After inhibition of NO-synthase, only responses to the lowest pH value were observed, while responses to moderate acidification were completely suppressed [23].

Acute metabolic acidosis in vivo (blood pH 7.2) was accompanied by a more prominent drop of mean arterial pressure to acetylcholine infusion compared to animals with a normal blood pH level [76]. Importantly, acute metabolic acidosis led to an increase in plasma NO metabolite levels, pointing to a higher NO production under acidic conditions in vivo [76].

### 3.2. Hypercapnic (Respiratory) Acidosis

Hypercapnic (respiratory) acidosis, like metabolic acidosis, may also be associated with an increased effect of NO on vascular tone. Several studies proved such an influence in the coronary vascular bed. Acute hypercapnia lasting 10 min induced by an increase in pCO_2_ from 38.6 mmHg to 61.4 mmHg (pH shift from 7.38 to 7.12) augmented coronary blood flow in isolated perfused mouse hearts; this effect was almost completely abolished in the presence of a NOS-inhibitor [77]. Qualitatively similar results were obtained in experiments with blood perfusion of the heart in anesthetized, open-chest dogs [78]. In this study, NOS inhibitors greatly suppressed dilatory responses of the coronary vasculature induced by hypercapnic acidosis (10% CO_2_, pH 7.16) and acetylcholine. In addition, endothelium-independent dilator responses of the coronary vasculature to sodium nitroprusside were resistant to NOS inhibitors, pointing to a larger effect of acidosis on the endothelium compared to its effect on the smooth muscle in the coronary vascular bed.

Further, the hypercapnia-induced increase in cerebral blood flow in rats was greatly reduced by a NOS inhibitor [79,80]. Accordingly, the relaxation responses to hypercapnic acidosis (10–15% CO_2_) were considerably reduced by inhibition of NO synthesis in rat small cerebral arteries: the basilar artery [81] and a branch of the middle cerebral artery [3]. The anticontractile effect of acidosis was almost three times smaller in the presence of a NOS inhibitor [3]. Along with that, blockade of NO-synthesis did not change the relaxatory responses to hypercapnic acidosis in experiments on isolated canine and monkey basilar and middle cerebral arteries [82]. Moreover, inhibition of NO synthesis blunted the effects of hypercapnic acidosis on blood flow in the human ocular artery [83] but not on the relaxatory responses of isolated porcine retinal arterioles [18]. Thus, the mechanisms of hypercapnic acidosis vary by animal species or by the experimental approach used. The reason for this is not yet clear.

### 3.3. Long-Term Effects of Acidosis on the eNOS Expression Level

Long-term effects of acidosis on the eNOS expression level were also observed, in addition to its acute effects on functional eNOS activity. Hypercapnic acidosis lasting 8 h augmented cerebral blood flow in anesthetized pigs. This effect was considerably reduced by treatment with a NO-synthase inhibitor [84]. In addition, hypercapnia caused an increase in cerebrovascular nitrite production associated with a rise in the brain eNOS mRNA expression level as well as an increase in NO-dependent vasorelaxant responses to substance P [84]. Similarly, increased levels of eNOS mRNA and protein were observed in the swine retina after 6–8-h long hypercapnia. This was associated with increases in retinal blood flow and NO metabolite production, both alterations were prevented by the administration of a NOS inhibitor [85]. The available data reporting long-term effects of metabolic acidosis on eNOS expression are controversial. On the one hand, metabolic acidosis induced by HCl infusion lasting 4 h caused a reduction in eNOS protein content in the lungs [86]. On the other hand, HCl-induced extracellular acidosis caused an increase in eNOS mRNA and eNOS protein content in human pulmonary arterial endothelial cells after 90 and 180 min, respectively [87].

Therefore, these data indicate that long-lasting acidosis not only affects eNOS activity but additionally it can change the expression of eNOS. This complex interplay between changes in eNOS activity and eNOS expression may result in different scenarios, either a synergistic effect when, e.g., eNOS activity and expression increase or an antagonistic effect when, e.g., eNOS activity increases and eNOS expression decreases. Due to the paucity of published data on this topic, it is unclear at this time whether there is a predominant scenario for most vessels or whether the scenarios depend on circumstances, e.g., the vessel bed affected. Further studies are required to get a deeper understanding of the long-term effects of acidosis on the functional impact of eNOS.

## 4. Effects of Acidosis on Mechanisms Controlling eNOS Activity

The data described above demonstrate that in many vascular beds acute extracellular acidosis-induced vasorelaxation is associated with increased eNOS functional activity. At the same time, direct in vitro measurements of eNOS activity indicate that acidosis causes a decrease in eNOS activity [87,88]. eNOS activity deduced from the conversion rate of L-[^14^C]arginine to L-[^14^C]citrulline in tissue homogenates or cell extracts was considerably reduced with acidification. Maximum activity of eNOS was observed at the physiologically relevant pH of 7.4 and activity was markedly decreased at pH values below 7.0 [87,88].

The apparent contradiction between the effects of acidosis on functional eNOS activity in intact cells, on the one hand, and on its enzymatic activity in homogenates, on the other hand, can be explained by the fact that intracellular Ca^2+^ and signaling pathways that regulate eNOS activity in intact cells are also dependent on pH [31,37].

Both metabolic and hypercapnic acidosis was shown to increase the intracellular Ca^2+^ concentration in cultured rat aortic endothelial cells [89]. Similarly, an increase in cytosolic Ca^2+^ was observed in human pulmonary artery endothelial cells under conditions of hypercapnic acidosis [90]. Moreover, intracellular acidification induced by Na^+^/H^+^-exchange inhibitors was accompanied by an increase in cytosolic Ca^2+^ in bovine aortic endothelial cells [91].

The TRPV4 channel is considered one of the channel types providing Ca^2+^ entry into endothelial cells and activation of eNOS [32,92,93]. Importantly, TRPV4 channel opening probability may increase in acidosis [94], thereby providing beneficial conditions for eNOS activation. Further, the current carried by another member of the TRP channel family, the TRPV1 channel, which is also implicated in Ca^2+^ entry into endothelial cells [32,39], increases under acidic conditions. Along with that, an increase in the intracellular Ca^2+^ concentration was still present in acidified endothelial cells after the removal of Ca^2+^ from the extracellular solution, pointing to the potentiating effect of acidosis on the release of Ca^2+^ from intracellular stores [89,90,91].

Importantly, acidosis was shown to affect the activity of kinases regulating eNOS functioning. Activation of signaling pathways involving CaMKIIα, PKA, and PI3/Akt-kinase under acidic conditions was previously reported in hippocampal slice culture [95]. For cardiovascular tissues, known effects of acidosis are limited to the PI3/Akt-kinase signaling pathway. In the densely vascularized myocardium, hypercapnic acidosis (20% CO_2_, pH 6.9) for the first 3 min of reperfusion after a 30-min long coronary occlusion reduced myocardial ischemia/reperfusion injury due to activation of the PI3/Akt/eNOS signaling pathway [96]. Such transient acidosis was associated with higher levels of Akt phosphorylation and, importantly, higher NO release from perfused hearts. All effects of acidosis were suppressed by wortmannin, a PI3-kinase inhibitor. Therefore, Akt activity may be increased in acidified coronary endothelial cells, and subsequently, the phosphorylation of eNOS at Ser1177, Ser633, and Ser615 and therefore eNOS activity should rise in acidosis. This assumption should be proven experimentally in future studies.

Contrarily, enzymatic and functional activities of Rho-kinase were shown to decrease during acidification in the vascular wall [27]. Therefore, the reduction of eNOS phosphorylation at the inhibitory site Thr495 might lead to an increase in eNOS functional activity in acidosis. On the other hand, currently known effects of acidosis on PKC activity are limited to non-vascular tissues, which complicates the analysis of its vasomotor effects in acidosis. An increased phosphorylation level and, presumably, the activity of PKC were observed in acidified intestinal mucosal cells [97]. In addition, PKC activated by acidosis was shown to increase the activity of Na^+^/H^+^-exchange in renal brush border membranes [98], which is favorable for the restoration of normal pH levels. However, the question about a PKC-dependent inhibition of eNOS in acidified endothelial cells remains open.

Thus, acidosis can affect the activity of several mechanisms, which would result in higher levels of eNOS functional activity. This may include increased Ca^2+^ entry into endothelial cells and Ca^2+^ release from intracellular stores, augmented phosphorylation of the activator sites via PI3/Akt-kinase, and reduced phosphorylation at the inhibitory site Thr495 via Rho-kinase. Presumably, acidosis-induced changes in these regulatory pathways may surpass the direct inhibitory effect of acidosis on eNOS enzymatic activity.

## 5. NO-Dependent Regulation of Vascular Tone in Early Ontogenesis

The early postnatal period of life in mammals is associated with intensive growth and development of many organs and tissues. This is also true for the vasculature, which undergoes remarkable structural and functional remodeling during the early postnatal period. At the systemic level, the cardiovascular system in early ontogenesis is characterized by a level of blood pressure that is almost two times lower than in an adult organism [99,100]. In addition, the distribution of cardiac output among organs is different in early postnatal ontogenesis compared to the adult organism [101]. For example, such organs as the skin and the gastrointestinal tract receive a large proportion of cardiac output in neonates and this proportion decreases considerably with maturation [101]. This is achieved by alterations in the mechanisms regulating vascular tone during early postnatal development.

Importantly, the NO-dependent mechanism of vascular tone regulation demonstrates high activity in neonates. For example, in the early postnatal period, many vascular regions of the systemic circulation possess a greater anticontractile influence of NO compared to the adult organism [30,102,103,104]. This is evident from a prominent augmenting effect of NOS inhibitors on arterial contractile responses at perinatal age compared to small effects of the inhibitors in adulthood [104,105]. The greater anticontractile influence of NO in early postnatal ontogenesis is associated with a higher eNOS expression [105,106]. In addition, the activity of the PI3/Akt/eNOS signaling pathway in the vascular endothelium may be increased in neonates which can provide a high functional activity of eNOS at this stage of postnatal development [105]. Accordingly, the activity of the NO/sGC/PKG pathway in arterial smooth muscle is also higher at perinatal age [105].

At the systemic level, a greater impact of endothelium-derived NO on the control of the circulatory system during early postnatal development is manifested in higher pressor responses to the NOS inhibitor L-NAME and higher blood content of NO metabolites compared to the adult organism [104,105,107]. Therefore, the anticontractile effect of NO can be an important mechanism responsible for maintaining the low blood pressure level in the immature circulatory system.

## 6. During Early Postnatal Development, Acidosis Reduces Systemic Blood Pressure Potentially Due to an Increase in eNOS Activity in the Arterial System

Of note, the data on the vascular effects of acidosis presented above were obtained on adult mature individuals, while the ontogenetic aspect of eNOS functioning under acidic conditions remained outside the research scope. Nevertheless, understanding the effects of pH on the regulation of vascular tone is important not only for an adult organism but also for an organism in the period of antenatal and early postnatal ontogenesis. Acidosis can occur in newborns due to neonatal hypoxia (asphyxia), which, unfortunately, often occurs during childbirth [108,109]. According to the World Health Organization reports, neonatal asphyxia is one of the most common causes of death among infants (about 30–35% of neonatal deaths) and its incidence varies by region, level of care, and other factors [110,111]. Severe acidosis (blood pH less than 7.0) in neonates is associated with a prominent blood pressure drop [111] but the role of NO in this response has not been explored, to the best of our knowledge. Notably, vasorelaxation in response to acidosis was shown to be greater in the newborn than in the adult rabbit aorta [112] and resistance mesenteric arteries [113].

**Figure 1:**
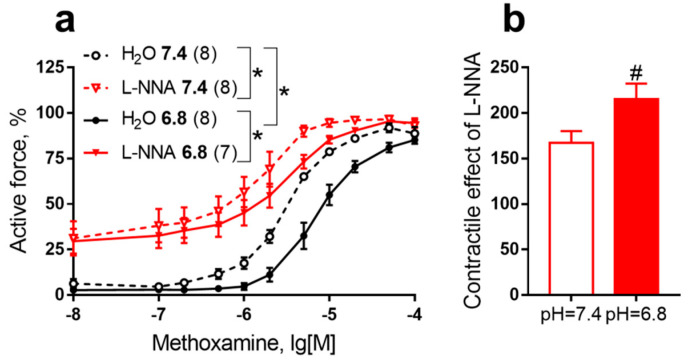
Acidosis potentiates the functional contribution of eNOS to the regulation of vascular tone in the early postnatal period. In 10–15-day old rat pups, extracellular acidosis (pH 6.8) reduces contractile responses of saphenous arteries to the α_1_-adrenoceptor agonist methoxamine by increasing the anticontractile influence of NO. (**a**) Concentration-response relationships to methoxamine in the presence of the NO-synthase inhibitor L-NNA (100 µM) or its vehicle (H_2_O, 50 µL) at pH 7.4 and pH 6.8. The numbers in brackets indicate the number of animals. * *p* < 0.05 (two-way ANOVA with Tukey’s multiple comparisons test). (**b**) Contractile effect of L-NNA at pH 7.4 and pH 6.8. # *p* < 0.05 (unpaired Student’s *t*-test).

To address the question of how acidosis in neonates affects the functional contribution of eNOS to the regulation of vascular tone, we conducted an experimental series to study the effect of metabolic acidosis (pH 6.8) on the anticontractile influence of NO in skin feed arteries (saphenous artery) of 10–15-day-old rat pups. Importantly, cutaneous blood flow accounts for up to 20% of cardiac output in rats during the first weeks of life [101] and, therefore, constriction or dilation of skin arteries can prominently affect the level of systemic blood pressure. The contribution of NO to the regulation of the cutaneous circulation is large in the early postnatal period and decreases with maturation [30,105]. Vasomotor responses of saphenous arteries were studied under in vitro conditions (for details on the research methods, see the Supplementary Materials). At pH 6.8 the contractile responses of saphenous arteries of 10–15-day-old rat pups to the α_1_-adrenoceptor agonist methoxamine were reduced compared to pH 7.4 (Figure 1a, black solid line vs. black dotted line). The NO-synthase inhibitor L-NNA augmented contractile responses to methoxamine both at pH 7.4 and at pH 6.8 (Figure 1a, pH 7.4: black dotted line vs. red dotted line; pH 6.8: black solid line vs. the red solid line). Therefore, NO exhibits its anticontractile influence both at normal pH and under acidic conditions. Notably, the reduction in the contractile responses to methoxamine induced by a change of pH from 7.4 to 6.8 in the absence of L-NNA (see the large distance between two black lines) was not observed when the same change in pH was performed in the presence of the NO-synthase inhibitor L-NNA (two red lines lie close to each other). In addition, the contractile effect of L-NNA was greater at pH = 6.8 compared to pH = 7.4 (Figure 1b). Therefore, the anticontractile influence of NO was increased by acidosis in 10–15-day old rat pups.

Thus, our data demonstrate that acidosis increases eNOS functional activity in arteries during early postnatal development, similar to its effect in the adult organism. Since the functional contribution of eNOS to the regulation of vascular tone during early postnatal ontogenesis is higher than in adulthood [30], the final effect of acidosis on total peripheral resistance in neonates may be stronger than in adults. The latter, however, should be proven in future studies. Of note, taking into account that acidosis often occurs during hypoxia in childbirth or during asphyxia [108], it can be assumed that this, among other things, will cause an increase in eNOS functional activity. Ultimately, this can lead to a decrease in peripheral resistance and hence blood pressure in infants. This response, in turn, may create additional health risks for neonates. In addition, acidosis-induced changes in eNOS functional activity, especially when accompanied by long-term alterations in eNOS expression as described in Section 3.3, may have ultra long-term effects appearing in adulthood. However, no studies are currently available addressing the question of whether acidosis-induced changes in eNOS functional activity are only temporary, reversible effects or may persist in adulthood (acidosis memory). Importantly, in the adult organism even more complexity is added by the fact that the direct effect of acidosis on vascular tone can be opposed by vasoconstriction resulting from activation of the chemoreflex [114,115]. In neonates, this compensation is limited by underdeveloped sympathetic control of the cardiovascular system [116,117]. Thus, further studies are required to get insight into possible consequences for an adult life of acidosis-induced changes in eNOS functional activity induced in the early postnatal period.

## 7. Conclusions

To conclude, published studies mostly indicate that the functional activity of the NO-pathway increases under acidic conditions, despite the direct effects of acidosis on eNOS enzymatic activity being rather inhibitory. This is possibly due to changes in signaling pathways regulating eNOS activity in endothelial cells. For example, acidosis may increase Ca^2+^ entry into endothelial cells through different types of TRP channels, induce Ca^2+^ release from intracellular stores, augment phosphorylation of the eNOS activator sites via PI3/Akt-kinase and reduce phosphorylation at the inhibitory site Thr495 via Rho-kinase. Importantly, acidosis potentiates the functional contribution of eNOS to the anticontractile impact of NO on vascular tone not only in adulthood but in the early postnatal period as well. Therefore, in conditions of neonatal asphyxia and acidosis, the NO influence might be one of the mechanisms causing an undesirable decrease in blood pressure in infants. Further detailed studies are needed to elucidate the systemic effects of acidosis on eNOS functional activity in the early postnatal period, considering the high incidence of acidosis in infants as well as possible long-term consequences for adult life.

## Data Availability

All data generated during this study are available from the corresponding author on reasonable request.

## Supplementary Materials

The following supporting information can be downloaded at: https://www.mdpi.com/article/10.3390/ijms23115987/s1. Reference [118] are cited in the Supplementary Materials.
