# Supplementary material for: The Effects of Acidosis on eNOS in the Systemic Vasculature: A Focus on Early Postnatal Ontogenesis
Source: Int J Mol Sci. 2022 May 26;23(11):5987. doi: 10.3390/ijms23115987 (PMC9180972; doi:10.3390/ijms23115987)
Supplement: Supplementary file 1 [file ijms-23-05987-s001.zip › ijms-1720828-supplementary.pdf]

SUPPLEMENTARY MATERIAL

The effects of acidosis on eNOS in the systemic vasculature: a focus on early postnatal ontogenesis

Dina K. Gaynullina[1], Olga S. Tarasova[1,2], Anastasia A. Shvetsova[1], Anna A. Borzykh[1,2], Rudolf Schubert[3]*

[1]Department of Human and Animal Physiology, Faculty of Biology, M.V. Lomonosov Moscow State University, 119234 Moscow, Russia; dina.gaynullina@gmail.com (D.K.G.); ost.msu@gmail.com (O.S.T.); anastasiashvetsova92@gmail.com (A.A.S.); borzykh.anna@gmail.com (A.A.B.)

[2]Laboratory of Exercise Physiology, State Research Center of the Russian Federation-Institute for Biomedical Problems, Russian Academy of Sciences, 123007 Moscow, Russia

[3]Physiology, Institute of Theoretical Medicine, Faculty of Medicine, University of Augsburg, 86159 Augsburg, Germany; rudolf.schubert@med.uni-augsburg.de

* Correspondence: rudolf.schubert@med.uni-augsburg.de

Experiments on the influence of acidosis on the anticontractile effect of NO were performed on 10-15-day-old male Wistar rats in accordance with the European Convention on the protection of animals used for scientific purposes (EU Directive 2010/63/EU). All animal procedures were approved by the Moscow State University committee on animal welfare (protocol 97-g-2, approval date 22.10.2021).

Saphenous arteries were isolated and carefully cleaned from surrounding tissue, cut into 2 mm long segments, and mounted in a wire myograph (410A or 620M, DMT A/S, Denmark) for isometric force recording. The isolation and mounting were performed in a physiological salt solution containing: NaCl, 145 mM; KCl, 4.5 mM; $CaCl_2$, 0.1 mM; $MgSO_4$, 1.0 mM; NaH_2PO_4, 1.2 mM; EDTA, 0.025 mM and HEPES, 5.0 mM (pH = 7.4). After mounting, the solution was replaced by the experimental solution, containing: NaCl, 120 mM; $NaHCO_3$, 26 mM; KCl, 4.5 mM; $CaCl_2$, 1.6 mM; $MgSO_4$, 1.0 mM; NaH_2PO_4, 1.2 mM; D-glucose, 5.5 mM; EDTA, 0.025 mM; and HEPES, 5.0 mM. The chambers of the myographs were heated to 37 °C and continuously bubbled with 5% CO_2 in O_2 to maintain pH = 7.4. Data were sampled at 10 Hz using an analogue-to-digital converter (E14-140M, L-CARD, Moscow, Russia) and PowerGraph 3.3 software (DISoft, Moscow, Russia).

At the beginning of each experiment the normalization procedure was performed in the low calcium solution supplemented with the NO-donor DEA/NO (1 μM), where each segment was stretched to 0.9*d100 (90% of the inner diameter the arterial segment would have at a transmural pressure of 100 mmHg) [1]. After the end of normalization, the solution was changed to the experimental solution and the following substances were applied to activate the preparation: (1) noradrenaline (10 μM, 5 min), (2) methoxamine (agonist of α_1-adrenoceptors, 3 μM, 5 min) followed by acetylcholine (10 μM, 2 min), to confirm the functional integrity of the endothelium; and (3) methoxamine (10 μM, 5 min).

Thirty minutes later, the first concentration-response relationship to methoxamine was performed (concentration range from 0.01 to 100 μM). After washout, in half of the preparations the solution was replaced with the experimental solution with pH=7.4 (the composition see above). In another half of the preparations the solution was replaced with acidic solution with pH=6.8 containing: NaCl, 140.5 mM; $NaHCO_3$, 5.5 mM; KCl, 4.5 mM; $CaCl_2$, 1.6 mM; $MgSO_4$, 1.0 mM;

NaH_2PO_4, 1.2 mM; D-glucose, 5.5 mM; EDTA, 0.025 mM; and HEPES, 5.0 mM. Ten minutes later the preparations were treated with the NO-synthase inhibitor L-NNA (100 μM) or an equivalent volume of its solvent (H_2O, 50 μL) for 20 min. Then the second concentration-response relationships to methoxamine were obtained (shown in Figure 1a).

To calculate active force values, the force value at the fully relaxed state was subtracted from all measured values. All values were expressed as percentage of maximum active force obtained during the first concentration-response relationship to methoxamine. In order to compare the anticontractile influence of NO at both pH values, areas under curves were calculated in GraphPad Prism 7.0 (La Jolla, CA, USA), after that, the areas in the absence of L-NNA were taken as 100% and finally we calculated by what percentage L-NNA increases the area under curves at pH=7.4 and at pH=6.8 (shown in Figure 1b).

Statistics were calculated using GraphPad Prism 7.0 (La Jolla, CA, USA). Data are shown as the mean ± S.E.M, n is the number of animals. Differences between groups were assessed using two-way ANOVA with Tukey's multiple comparisons test or Student's t-test and considered statistically significant at $p < 0.05$.

References

1. Mulvany, M.J.; Halpern, W. Contractile Properties of Small Arterial Resistance Vessels in Spontaneously Hypertensive and Normotensive Rats. *Circ. Res.* **1977**, *41*, 19–26.
